# Variations in national surveillance reporting for Mpox virus: A comparative analysis in 32 countries

**DOI:** 10.3389/fpubh.2023.1178654

**Published:** 2023-04-18

**Authors:** Deepkanwar Singh Panag, Nityanand Jain, Dimitra Katagi, Gabriela De Jesus Cipriano Flores, Gabriela Dias Silva Dutra Macedo, Gonzalo Rodrigo Díaz Villa, Mathieu Yèche, Saydi Yusveni Velázquez Mérida, Sreerag Kapparath, Zilfi Sert, Aigars Reinis

**Affiliations:** ^1^Faculty of Medicine, Riga Stradinš University, Riga, LV, Latvia; ^2^Faculty of Medicine, School of Medicine, University of Patras, Rio, Greece; ^3^Faculty of Medicine, Universidad Peruana Cayetano Heredia, Lima, Peru; ^4^Faculty of Medicine, Universidade da Região de Joinville (UNIVILLE), Joinville, Brazil; ^5^Pontifícia Universidade Católica do Paraná (PUCPR), Rua Imaculada Conceição, Prado Velho, Curitiba - PR, Brazil; ^6^Faculty of Medicine, Universidad de los Andes, Las Condes, Chile; ^7^ICM, Paris Brain Institute, Hopital de la Pitie-Salpetriere, Sorbonne Universite, INSERM U1127, CNRS UMR7225, Paris, France; ^8^Département de Biologie de l’École Normale Supérieure (ENS), PSL Research University, Paris, France; ^9^Faculty of Human Medicine “Manuel Velasco Suárez”, Universidad Autónoma de Chiapas, Tapachula, Chiapas, Mexico; ^10^Faculty of Medicine, Tbilisi State Medical University, Tbilisi, Georgia; ^11^Faculteit der Bètawetenschappen, Vrije Universiteit Amsterdam, Amsterdam, Netherlands; ^12^Joint Laboratory, Pauls Stradinš Clinical University Hospital, Riga, Latvia

**Keywords:** case definitions, differences, epidemiology, Monkeypox, mpox, reporting, surveillance

## Abstract

**Objectives:**

Case Reporting and Surveillance (CRS) are crucial to combat the global spread of the Monkeypox virus (Mpox). To support CRS, the World Health Organization (WHO) has released standardized case definitions for suspected, probable, confirmed, and discarded cases. However, these definitions are often subject to localized adaptations by countries leading to heterogeneity in the collected data. Herein, we compared the differences in Mpox case definitions in 32 countries that collectively reported 96% of the global Mpox caseload.

**Methods:**

We extracted information regarding Mpox case definitions issued by the competent authorities in 32 included countries for suspected, probable, confirmed, and discarded cases. All data were gathered from online public sources.

**Results:**

For confirmed cases, 18 countries (56%) followed WHO guidelines and tested for Mpox using species specific PCR and/or sequencing. For probable and suspected cases, seven and eight countries, respectively were found to have not released definitions in their national documentations. Furthermore, none of the countries completely matched WHO’s criteria for probable and suspected cases. Overlapping amalgamations of the criteria were frequently noticed. Regarding discarded cases, only 13 countries (41%) reported definitions, with only two countries (6%) having definition consistent with WHO guidelines. For case reporting, 12 countries (38%) were found to report both probable and confirmed cases, in line with WHO requirements.

**Conclusion:**

The heterogeneity in case definitions and reporting highlights the pressing need for homogenization in implementation of these guidelines. Homogenization would drastically improve data quality and aid data-scientists, epidemiologists, and clinicians to better understand and model the true disease burden in the society, followed by formulation and implementation of targeted interventions to curb the virus spread.

## 1. Introduction

In the domain of public health, Case Reporting and Surveillance (CRS) is a quintessential component in controlling the spread of communicable diseases in society. CRS enables real-time monitoring of the spread of the disease-causing pathogen not only within a specific community but also at a global level. Such surveillance allows for evaluation and prediction of the course of the disease, while contributing toward the formulation of targeted interventions to truncate the spread (1). Additionally, it enables the identification of the most vulnerable population groups (e.g., the older adult population in initial COVID-19 waves) (2), thereby facilitating moderation and diversion of the already scarce healthcare resources to those most in need.

Accordingly, the International Health Regulations (IHR) 2005, were adopted in 2007 by 196 countries and serve as a legally binding instrument that empowers the World Health Organization (WHO) as the main global surveillance system and requires the WHO to declare certain pathogens as public health emergency of international concern (PHEIC) (3). PHEIC is any hazard (radiological, chemical, and biological) that has the potential of international spread and requires an immediate and coordinated global response. The IHR 2005 require all signatory countries to develop and maintain the capacity to detect, assess, report, and respond to PHEIC(s). Despite the adoption of IHR 2005 more than a decade ago, the Centers of Disease Control and Prevention (CDC) estimates that about 2/3rd of the countries lack such capacities, thereby leaving the world population vulnerable (4). In the past, countries with potential violations of the IHR 2005 have gone by without suffering many serious consequences (5), attributable mostly to the lack of guidelines about the mandatory dispute settlement process or enforcement mechanism (5).

To aid the member countries, the WHO regularly releases interim guidelines and other relevant documentation for proper recording, isolation, and reporting of suspected, probable, and confirmed cases. However, experiences from previous epidemics and pandemics have highlighted that there is a lack of standardization when it comes to the international implementation of case definitions and surveillance guidelines (6). Taking the recent example of COVID-19, Suthar et al., found that in the 25 most affected countries, only 56% of the countries followed WHO’s recommended case definition for suspected cases (7). Similar findings were seen for probable and confirmed cases (7). This is not the first time such heterogeneity has been described (8, 9). In the European Union (EU), a review of maritime hygiene and disease control manuals also found variances in disease surveillance practices and called for a need for the implementation of common rules and tools (10).

Implementation and adherence to standardized case definitions allow for easy intra- and inter-country reporting and compilation of data, thereby enhancing the data quality. Such practices also allow for the maximum inclusion of population characteristics which is essential when preparing disease models. A test conducted by Krause et al., in Germany allowed the authors to analyze intra-country variations in the implementation of case definitions (11). The author’s work proved to be instrumental in overhauling the case reporting system in Germany and allowed for inclusive and more realistic reporting (11). Several calls in the past have been raised for the WHO to step up and homogenize the case-reporting hierarchy, however, differences in technical and financial capabilities in the lower- and middle-income countries (LMICs) often limit this exercise (6).

The latest biological PHEIC to be classified is the Monkeypox Virus (Mpox), a zoonotic virus endemic to the rainforests of central and western Africa (12). The virus has spread rapidly in the Global North and has been detected in more than 100 countries as of 30th January 2023. In response, the WHO released standardized case definitions (13) and two separate forms for case investigation (CIF) and case reporting (CRF) (14). The CIF is meant for in-depth epidemiological investigations while the CRF is a minimum dataset capturing the key epidemiologic parameters of monkeypox cases. Currently, the WHO mandates member states to submit the CRF for probable and confirmed cases under Article 6 of the IHR 2005 (13).

Given the possibility of differences in the implementation of WHO recommendations based on previous experiences, we henceforth, undertook the present study whereby we investigated how different countries adopted WHO’s Mpox guidelines and recommendations in terms of case definitions and case notification. We believe the results obtained in the present study would be useful to the international community at large, given the current spread of Mpox. Highlighting such discrepancies at earlier stages of the disease spread could potentially aid in proper implementation, capacity building, and updating of the respective national guidelines.

## 2. Methods

In the present study, we investigated data from 32 countries. These countries were included based on the following criteria: (i) most affected countries (the countries with the highest Mpox caseload); (ii) publicly available information on Mpox case definitions; (iii) release of such definitions by the country’s competent authority and (iv) access to the information. We additionally, excluded countries that are considered endemic and/or had been known historically to be the source of outbreaks. Accordingly, we included 17 out of 20 of the most affected countries along with 15 other countries with relatively low Mpox caseloads. The other three countries in the top 20 most affected countries—Nigeria, Democratic Republic of Congo, and Ecuador were not included due to historically known outbreaks in the first two and no case definition information found for the latter.

Information regarding Mpox case definitions for suspected, probable, confirmed, and discarded cases were collected and gathered from online public sources. Additionally, we collected information regarding which of the Mpox cases are required to be notified to the Health Officers/National Reporting Systems (NRS) by healthcare practitioners. For the data that was not available in English, for the following languages, the data was validated by the authors (native language speakers)—Dutch, French, Greek, Portuguese, Spanish, and Turkish. For other languages, we used bi-directional online language conversion (using Google Translate)—first from the language of the official document to English and then in the reverse direction (for accuracy). A list of sources used for each of the investigated countries along with full case definitions and their translations are provided in Supplementary File.

The investigated Mpox guidelines and documents were “current and in effect” in the respective countries as of January 2023 (based on available and accessible online data; there may be newer versions which are not immediately available online). The sources were last checked for updates on 25th January 2023. The study design was restricted to observational, cross-sectional, qualitative comparison (including descriptive statistics) and inferential statistical analysis was not undertaken due to the nature of the collected data. The STROBE checklist was used for reporting the data. Since the data analyzed are available publicly, ethical approval did not apply to the present study.

## 3. Results

### 3.1. Spread of Mpox across the investigated countries

**Figure 1:**
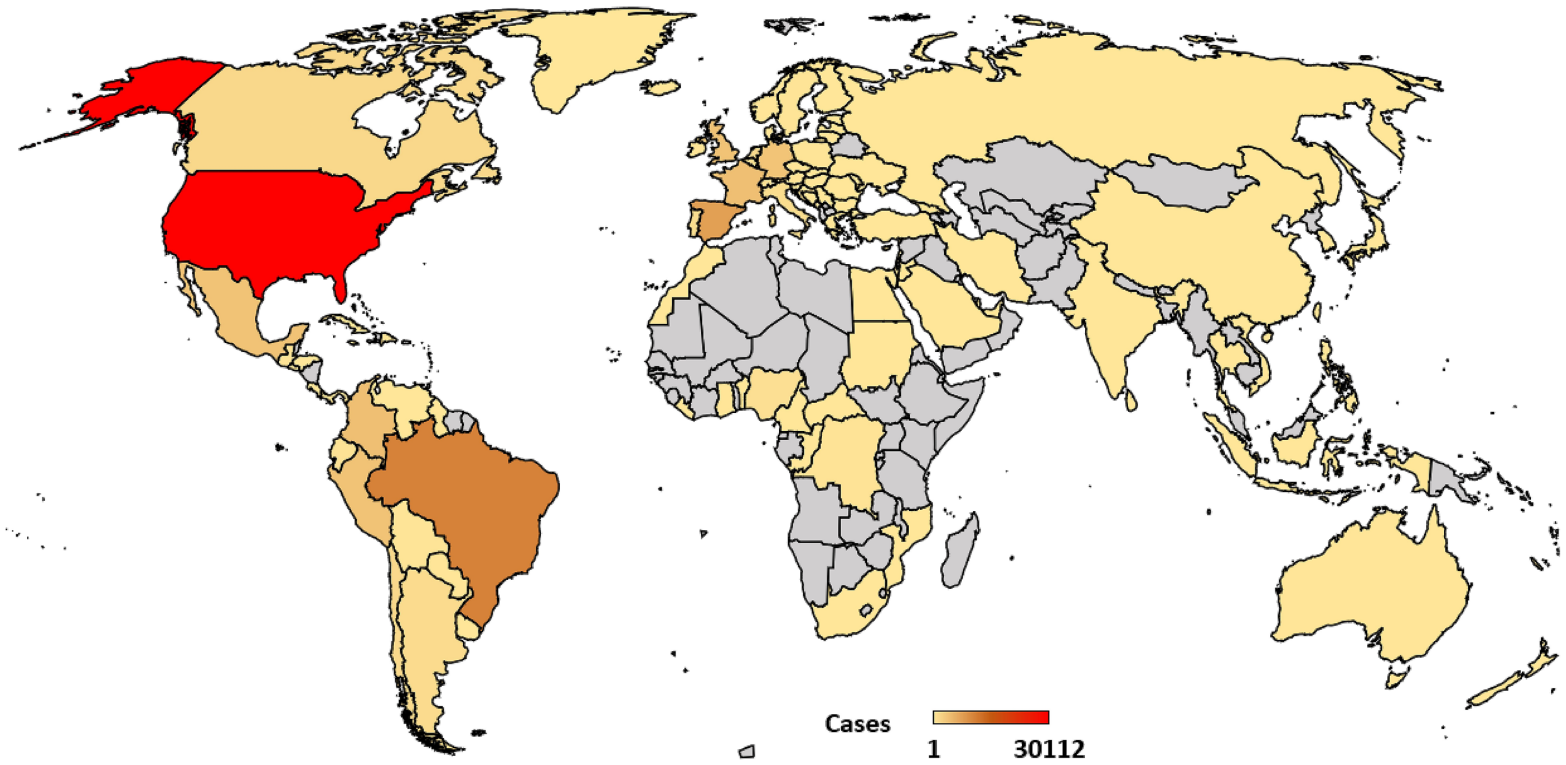
Geographical distribution of Mpox caseload as of January 31, 2023 (confirmed cases only). Data source—United States Centers for Disease Control and Prevention (U.S. CDC; https://www.cdc.gov/poxvirus/monkeypox/response/2022/world-map.html; accessed February 1, 2023). Note that the map is only for illustrative purposes and the authors remain neutral regarding territorial disputes.

Since May 2022, more than 81,000 cases of Mpox have been confirmed as of 31st January 2023 in the investigated 32 countries, representing almost 96% of the total cases reported globally (Figure 1). The United States of America (USA) reported the highest caseload (35.2%) followed by Brazil and Spain (12.6 and 8.8%, respectively). Together, these three countries account for more than 55% of all confirmed cases globally. Furthermore, nine of the 32 included countries reported Mpox-related deaths (70 deaths combined), accounting for 63% of all reported Mpox-related deaths worldwide. The highest number of deaths were recorded in the United States (27) followed by Brazil and Peru (15 each). India and Belgium each reported 1 Mpox-related death.

### 3.2. Case definitions for laboratory confirmed cases

The WHO’s guidelines for the classification of confirmed Mpox case requires the detection of unique sequences of the Mpox DNA using either (i) polymerase chain reaction (PCR) or (ii) sequencing (Table 1). Furthermore, PCR conducted on blood samples is not considered diagnostically sufficient and should not be used as a stand-alone first-line diagnostic tool. The primary sampling for PCR should be done from the skin lesion material or other specimen such as an oral or nasopharyngeal swab. We observed that all countries required molecular testing with PCR as a pre-condition for classification of cases as “Mpox positive.” Noticeably, 11 countries also accepted generalized Orthopoxvirus detection by Nucleic Acid Amplification Tests (NAATs) which may or may not be followed up by Mpox specific sequencing. Furthermore, only 18 out of 32 countries mentioned sequencing as a criterion for confirmed cases. Australia and Sweden also accepted isolation of virus in culture (represents one of the most precise techniques to demonstrate active reproduction of the virus) as a criterion for confirmed cases. Germany, on the other hand, accepted positive electron microscopy results as an additional criterion.

**Table 1:** Criteria for defining Mpox confirmed case in the investigated countries.

Country	Detection of unique sequences of Mpox DNA using		Isolation of Mpox in culture
	Polymerase chain reaction (PCR)	Sequencing	
World Health Organization (WHO)	X	X	
Argentina^1^^,^^2^	X		
Australia^3^	X	X	X
Austria^3^^,^^4^	X	X	
Belgium^5^	X		
Brazil^2^	X	X	
Canada	X	X	
Chile^6^	X		
Colombia^7^^,^^8^	X		
Costa Rica	X	X	
Cyprus^4^	X	X	
Denmark	X		
France^9^	X		
Germany^9^^,^^10^	X	X	
Greece^4^	X	X	
India	X	X	
Ireland	X		
Italy^6^	X	X	
Jamaica^6^	X	X	
Mexico	X	X	
Netherlands^8^	X		
New Zealand^3^	X		
Peru^3^^,^^7^	X		
Poland^4^	X	X	
Portugal	X	X	
South Africa^6^	X	X	
Spain^9^	X		
Sweden^3^	X	X	X
Switzerland	X		
Turkiye^7^	X		
UAE^6^	X	X	
United Kingdom	X		
United States	X	X	
Total	32/32 (100%)	18/32 (56%)	2/32 (6%)

1 Argentina also accepts detectable PCR results for Orthopoxvirus if patient belongs to the Eurasian-African group.

2 Argentina and Brazil require patients to meet criteria for suspected case along with laboratory confirmation to be classified as confirmed case.

3 Australia, Austria, New Zealand, Peru, and Sweden does not specify PCR in guidelines but require patients to perform any Nucleic Acid Amplification Test (NAAT) or molecular testing.

4 Austria, Cyprus, Greece, and Poland also accept a positive PCR result for Orthopoxvirus if it is followed by sequencing showing Mpox in a person who developed symptoms after March 01, 2022, onwards.

5 Belgium also accepts Orthopoxvirus specific PCR assay positive result in patients with symptom onset after March 1, 2022.

6 Chile, Italy, Jamaica, South Africa, and UAE require patients to meet criteria for either suspected or probable case along with laboratory confirmation to be classified as confirmed case.

7 Colombia, Peru, and Turkiye requires patients to meet criteria for probable case along with laboratory confirmation to be classified as confirmed case.

8 Colombia and Netherlands accept Orthopoxvirus specific PCR without follow-up sequencing.

9 France, Germany, and Spain, in addition to Mpox specific PCR, also accept Orthopoxvirus specific PCR.

10 Germany also accepts results of Electron microscopy as criterion for confirmed case.

### 3.3. Case definitions for probable cases

According to WHO, an individual is classified as a probable case of Mpox if the individual meets the clinical criteria along with one or more of several additional criteria (Table 2). The clinical criteria require that an individual present with either an unexplained acute rash, mucosal lesions, or lymphadenopathy. There are five additional criteria described by WHO for the probable case definition—(i) epidemiologic link to a probable or confirmed case in the 21 days prior to symptom onset; (ii) identified as gay, bisexual, or other man who has sex with men; (iii) multiple or casual sexual partners in the 21 days prior to symptom onset; (iv) detectable levels of anti-orthopoxvirus (OPXV) IgM or IgG antibody titers; and (v) positive test result for orthopoxvirus infection.

**Table 2:** Criteria for defining Mpox probable (or likely) case in the investigated countries.

Country	Clinical Criteria			Additional Criteria (must fulfill one or more along with clinical criteria)					
	Unexplained acute skin rash^1^	Mucosal lesions^2^	Lymphadenopathy	Epidemiological link^3^	Travel to endemic regions^4^	Identifies as gay, or MSM community member	Multiple sexual partners^5^	Detectable IgM or 4x rise in IgG^6^	Positive for OPXV infection^7^
World Health Organization (WHO)	X	X	X	X		X	X	X	X
Australia^a^	X	X	X						X
Austria^b^	X	X	X	X	X		X		X
Belgium^c^	X		X	X	X	X	X		
Brazil^d^^,^^e^	X	X		X			X		
Canada^e^^,^^f^	X	X		X					
Chile^d^^,^^g^^,^^h^	X		X	X	X				
Colombia^i^	X	X	X	X	X		X		
France^e^^,^^g^^,^^j^	X	X	X	X					
Germany^k^	X	X	X	X					
India^d^^,^^e^^,^^f^^,^^h^	X		X	X					
Ireland^l^	X	X	X	X	X				
Italy^d^^,^^e^^,^^l^^,^^m^	X		X	X	X		X		X
Jamaica^d^^,^^e^^,^^f^^,^^l^	X		X	X	X				
Mexico^l^	X	X	X						
Netherlands^l^^,^^n^	X	X	X	X		X	X		
New Zealand^e^	X	X		X	X	X	X		
Peru^e^^,^^f^^,^^o^	X		X	X			X		
Poland^e^^,^^l^	X		X	X	X	X	X		X
Portugal^d^^,^^e^^,^^m^^,^^p^	X	X	X	X	X		X		
South Africa^d^^,^^e^^,^^l^^,^^m^	X		X	X	X		X		X
Spain^l^	X		X	X	X	X	X		
Switzerland^d^^,^^q^	X			X					
UAE^d^^,^^r^	X		X	X	X		X		
United Kingdom	X	X		X		X	X		
United States^s^								X	X
Total	24/25 (96%)	13/25 (52%)	19/25 (76%)	22/25 (88%)	13/25 (52%)	6/25 (24%)	14/25 (56%)	1/25 (4%)	6/25 (24%)

1 The skin rash may include single or multiple lesions in the ano-genital region or elsewhere on the body.

2 Mucosal lesions may include single or multiple oral, conjunctival, urethral, penile, vaginal, or ano-rectal lesions. Anorectal lesions can also manifest as ano-rectal inflammation (proctitis), pain and/or bleeding.

3 The person has been exposed to a probable or confirmed monkeypox case in the 21 days before symptom onset. For countries with animal to human transmission, known contact with wild animals (dead or alive) and/or sick animals in the 21 days before the onset of symptoms is included as epidemiological criteria.

4 The person has traveled to Mpox endemic African regions in the 21 days before symptom onset [Benin, Cameroon, the Central African Republic, the Democratic Republic of the Congo, Gabon, Ghana (identified in animals only), Ivory Coast, Liberia, Nigeria, the Republic of the Congo, Sierra Leone, and South Sudan].

5 The person has had multiple and/or casual sexual partners in the 21 days before symptom onset.

6 The person has detectable levels of anti-orthopoxvirus (OPXV) IgM antibody (during the period of 4–56 days after rash onset); or a 4-fold rise in IgG antibody titer based on acute (up to day 5–7) and convalescent (day 21 onwards) samples; in the absence of a recent smallpox/monkeypox vaccination or other known exposure to OPXV. Note that serology is not first line diagnostic modality for Mpox and should be used for retrospective case classification when PCR skin lesion testing was not possible or for research purposes.

7 The person has a positive test result for orthopoxviral infection done on a sample other than blood specimen (e.g., OPXV-specific PCR without Mpox-specific PCR or sequencing).

a Australia also accepts detection of orthopoxvirus by electron microscopy from clinical specimens in the absence of exposure to another orthopoxvirus. Additionally, in clinical criteria, Australia also mentions history of fever (>38°C), headache, myalgia, arthralgia, fatigue, and back pain.

b Austria in clinical criteria additionally accepts fever (>38.5°C), myalgia, arthralgia, cephalgia, back pain, painful lymphadenopathy (localized or generalized), and fatigue (prodromal stage). Additionally, contact with rodents or non-human primates in or from affected areas that allows animal-to-human transmission and occupational exposure to smallpox viruses are also considered as criteria in the case definition.

c Belgium also considers following symptoms for Mpox infection—fever (usually high >38.5°C), headache, back ache, and fatigue.

d Brazil, Chile, India, Italy, Jamaica, Portugal, South Africa, Switzerland, and UAE requires probable cases to meet the definition of suspected cases.

e Brazil, Canada, France, India, Italy, Jamaica, New Zealand, Peru, Poland, Portugal, and South Africa also consider prolonged and close exposure without respiratory protection and contact with contaminated materials, such as bedding and bath linen or utensils for common use belonging to a probable or confirmed case within the last 21 days of symptom onset as criteria for probable case. Healthcare workers meeting suspected case definition and improperly using personal protective equipment who got in contact with probable or confirmed case are also considered as probable case.

f Canada, India, Jamaica, and Peru considers travel history to or residence in a location where monkeypox is reported in the last 21 days of symptom onset as criteria for epidemiological link.

g Chile and France for epidemiological criteria requires contact with confirmed cases only.

h Chile and India additional clinical symptoms included in case definition include headache, sudden onset of fever (>38.5°C), myalgia, back pain, and asthenia (weakness).

i Colombia also considers following symptoms in clinical criteria—fever, sore throat, myalgia, and headache. Additionally, Colombia considers contact with live or dead animal potential reservoirs of the virus in African region as epidemiological criteria. It also considers travel to countries with confirmed outbreak as another criteria.

j France additionally considers fever (>38°C) and odynophagia as clinical criteria. In epidemiological criteria, France also considers unprotected contact <2 m for ≥3 h with probable or confirmed symptomatic case.

k Germany refers to probable cases as “Clinically-epidemiologically confirmed disease.” Clinical criteria also include fever, and disease-related death.

l Ireland, Italy, Jamaica, Mexico, Netherlands, Poland, Spain, and South Africa considers following clinical symptoms in probable case definition–-acute illness with fever (>38.5°C), headache, myalgia, arthralgia, back pain, and asthenia.

m Italy, Portugal, and South Africa also considers any hospitalized person due to Mpox-like illness as probable case.

n Netherlands also considers a female partner of a man who (also) has sex with men as epidemiological criteria. Netherlands refers to probable cases as “Likely case.”

o Peru also considers following clinical symptoms in clinical criteria—fever, fatigue, muscular pain, vomiting, diarrhea, shaking chills, throat pain, and headache.

p Portugal also considers sudden onset fever (≥38.0°C), asthenia, myalgia, back pain, and headache as clinical symptoms. Portugal uses definition of high-risk contacts for all epidemiological contacts.

q Switzerland considers either fever (or flu like symptoms) with acute rash or fever or rash with epidemiological link as definition for suspected and probable case.

r UAE considers acute rash interchangeable with fever (>38.5°C) and requires at least two or more of the following clinical symptoms to be present in the patient—headache, myalgia (muscle pain/body aches), back pain, and asthenia.

s United States characterizes probable case as case with no suspicion of other recent Orthopoxvirus exposure (e.g., Vaccinia virus in ACAM2000 vaccination) and no laboratory evidence of infection with another non-variola orthopoxvirus. Further, demonstration of orthopoxvirus DNA by molecular testing of a clinical specimen or orthopoxvirus using immunohistochemical or genomic sequencing testing methods is required for probable case definition.

Seven of the investigated countries (Argentina, Costa Rica, Cyprus, Denmark, Greece, Sweden, and Turkiye) did not report case definitions for probable cases. Among the remaining countries, presence of unexplained rash in any age group patient after March 1, 2022, and high-risk contact with confirmed or probable case were noted as the most common criteria (Table 2). More than half of the countries also included an additional criterion (when compared with WHO’s guidelines) of travel to endemic regions in Africa in the last 21 days of symptom onset. Only the United States uses serological criteria for probable cases. For countries with animal to human transmission, WHO states that known contact with wild animals (dead or alive) and/or sick animals in the 21 days before the onset of symptoms should be included in the epidemiological criteria. Austria and Colombia were the only two countries to issue guidelines in this regard.

Interestingly, only six countries—Belgium, Netherlands, New Zealand, Poland, Spain, and United Kingdom—included identification of patient as gay or MSM (men who have sex with other men) community member as a criterion in the case definition of probable cases. Netherlands also considers a female partner of a man who (also) has sex with men as epidemiological criteria. Both Poland and New Zealand also included prolonged and close exposure without respiratory protection and contact with contaminated materials, such as bedding and bath linen or utensils for common use belonging to a probable or confirmed case within the last 21 days of symptom onset as criteria for probable case. Healthcare workers meeting suspected case definition and improperly using personal protective equipment who got in contact with probable or confirmed case were also considered as probable case in these two countries.

Australia additionally accepts electron microscopy results for defining probable cases (while Germany used the technique for confirmed cases). United States, on the other hand, accepts immunohistochemistry and genomic sequencing results for probable case definitions. Application of diagnostic electron microscopy (EM) can provide initial results within minutes and can successfully aid in excluding majority of differential diagnosis (15). However, it must be followed by more specific tests since EM cannot identify different viral species.

### 3.4. Case definitions for suspected cases

The WHO defined a suspected case as an individual fulfilling either of the two criteria. The first criteria included epidemiological contact with a confirmed or probable Mpox case in the 21 days before the onset of signs or symptoms and who presents with any of the following—acute onset of fever (>38.5°C), headache, myalgia (muscle pain/body aches), back pain, profound weakness, or fatigue. The second criteria included clinical criteria (unexplained acute skin rash, mucosal lesions, or lymphadenopathy) and testing criteria (exclusion of other common causes of acute rash or skin lesion and testing for Mpox in case of co-infections).

We found that eight countries did not report case definitions for suspected cases (Austria, Colombia, Germany, Ireland, Mexico, New Zealand, Peru, and Poland). Austria previously defined suspected cases separately but in the updated 2023 guidelines, the country defines suspected and probable cases singly as probable cases. Similarly, Mexico removed the definition of suspected cases in the updated August 2022 guidelines. Though the exact rational is not immediately clear behind these changes, we suspect these changes would streamline the reporting process (in line with WHO recommendations) and provide for a binary classification of cases for the medical personnel.

Interestingly, New Zealand has a classification category called “Under Investigation” that is defined as a person that has been reported to a Medical Officer of Health, but information is not yet available to classify it as confirmed, probable or not a case. Among the other countries, unexplained acute skin rash and fever were found to be the most used clinical symptoms, followed by lymphadenopathy and headache (Table 3). Belgium and Brazil were the only country (in line with WHO guidelines) that recommended testing for Mpox in highly suspicious cases in whom an alternative pathogen has been identified to check for co-infections.

**Table 3:** Criteria for defining Mpox suspected (or possible) case in the investigated countries.

Country	Criteria 1						Criteria 2				
	Epidemiological criteria^1^	Clinical criteria					Clinical criteria^2^			Testing criteria	
		Acute onset Fever > 38.5°C	Headache	Myalgia	Back pain	Profound weakness or fatigue	Unexplained acute skin rash	Mucosal lesions	Lymphadenopathy	Exclude other causes of rash^3^	Testing for Mpox in case of co-infections^4^
World Health Organization (WHO)	X	X	X	X	X	X	X	X	X	X	X
Argentina ^a^	X	X	X	X	X	X	X	X	X	X	
Australia ^b^^,^^c^	X	X	X	X	X	X	X	X	X		
Belgium ^d^		X	X	X	X	X	X		X		X
Brazil ^d^							X	X		X	X
Canada ^e^		X	X	X	X	X	X	X	X		
Chile ^d^		X	X	X	X	X	X		X	X	
Costa Rica ^a^^,^^c^^,^^f^	X	X	X	X	X	X	X	X	X	X	
Cyprus ^c^^,^^d^^,^^g^	X	X	X		X	X	X		X		
Denmark ^c^	X	X	X		X	X	X	X	X		
France ^c^^,^^h^		X					X	X	X		
Greece ^c^^,^^d^^,^^g^	X	X	X		X	X	X		X		
India ^d^^,^^i^		X	X	X		X			X		
Italy		X	X	X	X	X	X		X	X	
Jamaica		X	X	X	X	X	X		X	X	
Netherlands		X	X	X	X	X	X	X	X	X	
Portugal ^j^		X	X	X	X	X	X	X	X	X	
South Africa		X	X	X	X	X	X		X	X	
Spain ^k^		X	X	X	X		X		X	X	
Sweden ^l^	X										
Switzerland	X	X					X				
Turkiye ^k^^,^^m^	X	X	X	X		X	X		X		
UAE		X	X	X	X	X	X		X		
United Kingdom ^j^^,^^k^	X	X	X	X	X	X	X	X	X		
United States ^c^^,^^d^^,^^n^	X	X					X	X	X	X	
Total	11/24 (46%)	22/24 (92%)	19/24 (80%)	16/24 (67%)	17/24 (71%)	18/24 (75%)	22/24 (92%)	11/24 (46%)	21/24 (88%)	11/24 (46%)	2/24 (8%)

1 A person who is a contact of a probable or confirmed monkeypox case in the 21 days before the onset of signs or symptoms. For countries with animal to human transmission, known contact with wild animals (dead or alive) and/or sick animals in the 21 days before the onset of symptoms is included as epidemiological criteria.

2 A person presenting since 01 January 2022 with an unexplained acute skin rash, mucosal lesions, or lymphadenopathy (swollen lymph nodes). The skin rash may include single or multiple lesions in the ano-genital region or elsewhere on the body. Mucosal lesions may include single or multiple oral, conjunctival, urethral, penile, vaginal, or ano-rectal lesions. Ano-rectal lesions can also manifest as ano-rectal inflammation (proctitis), pain and/or bleeding.

3 Differentials include varicella zoster, herpes zoster, measles, herpes simplex, bacterial skin infections, disseminated gonococcus infection, primary or secondary syphilis, chancroid, lymphogranuloma venereum, granuloma inguinale, molluscum contagiosum, allergic reaction (e.g., to plants); and any other locally relevant common causes of papular or vesicular rash. It is not necessary to obtain negative laboratory results for listed common causes of rash illness in order to classify a case as suspected.

4 If suspicion of monkeypox infection is high due to either history and/or clinical presentation or possible exposure to a case, the identification of an alternate pathogen which causes rash illness should not preclude testing for Mpox, as co-infections have been identified.

a Argentina and Costa Rica additionally considers contact with contaminated materials—such as clothing or bedding used by suspected or confirmed case, close contact without respiratory protection, and sexual intercourse with multiple partners in the past 21 days of symptom onset as epidemiological criteria.

b Australia considers fever >38°C or history of fever as criteria for clinical evidence along with extra symptom of arthralgia.

c Australia, Costa Rica, Cyprus, Denmark, France, Greece, and United States additionally considers overseas travel (especially to endemic regions) in the 21 days before symptom onset and sexual contact and/or other physical intimate contact with multiple partners (of any orientation) and/or a gay, bisexual, or other man who has sex with men in the 21 days before symptom onset as epidemiological criteria.

d Belgium, Brazil, Chile, Cyprus, Greece, India, and United States define acute onset rash as unexplained generalized or localized maculopapular or vesiculopustular rash with centrifugal spread, with lesions showing umbilication or scabbing and progressing through following stages—macules, papules, vesicles, pustules, and crusts.

e Canada defines rash as progressively developing that usually starts on the face and then spreads elsewhere on the body. The rash can affect the mucous membranes in the mouth, tongue, and genitalia. The rash can also affect the palms of hands and soles of the feet. The rash can last for 2—4 weeks and progresses through the following stages before falling off: macules, papules, vesicles, pustules, and scabs.

f Costa Rica also considers medical history of recent consultations for suspected STI diseases as epidemiological criteria.

g Cyprus and Greece also considers positive result in a test for the detection of viruses of the genus Orthopoxvirus as criteria for suspected case classification.

h France defines case with clinical presentation as “suspected case” but a case with clinical presentation and high-risk contact (refer to footnote point c above) as “possible case.”

i India considers travel to affected countries in the past 21 days of symptom onset as epidemiological criteria.

j Portugal and United Kingdom considers fever as ≥38°C.

k Spain, Turkiye, and United Kingdom additionally consider arthralgia as a clinical symptom suggestive of Mpox. For United Kingdom only, chills are also an additional symptom.

l Sweden considers suspected case as a person sampled with suspicion of monkeypox pending test results and who has no symptoms but has fulfilled the epidemiological criteria.

m Turkiye defines rash as itchy lesion without specific differentiation of the type of lesion (mucosal or dermal).

n United States also considers contact with a dead or live wild animal or exotic pet that is an African endemic species or used a product derived from such animals as epidemiological criteria. Additionally, contact, without the use of appropriate PPE or Biosafety Level (BSL) protocols, with laboratory specimens or other items or contact without appropriate use of PPE with a person or animal with a known orthopoxvirus or Mpox virus infection are considered in the criteria.

For epidemiological criteria, only 46% of the countries fulfilled WHO criteria. For suspected cases, United States was the only country to include contact with infected animal as one of the many possible epidemiological criteria. Interestingly, many countries did not clearly divide the criteria for suspected case in the manner prescribed by WHO. Overlapping amalgamations of the two criteria were seen in most countries (refer to Supplementary File for individual country definitions).

### 3.5. Notifiable cases

According to the WHO, national authorities should collect data for all cases that meet the case definitions for probable and confirmed cases. The data pertaining to suspected cases should be maintained at national level. Accordingly, we found that 12 countries (38%) followed the WHO guidelines, with seven countries asking medical practitioners to additionally notify suspected cases (Table 4). Germany, Netherlands, Sweden, and Switzerland require notification for only confirmed cases while nine countries (28%) required notification of confirmed and suspected cases. Noticeably, Netherlands, downgraded reporting of cases from group A (confirmed and probable cases) to group B1 (only confirmed cases) as of 15th December 2022.

**Table 4:** Differences in notifiable cases based on surveillance definitions.

Notifiable cases based on surveillance definitions	Number of countries	Countries
Confirmed cases only	4	Germany^1^, Netherlands, Sweden, and Switzerland
Confirmed and probable cases (WHO recommended)	12	Australia^2^, Austria, Canada, Colombia, France^3^, Ireland, Mexico, New Zealand, Peru, Poland^4^, United States, and United Kingdom^5^
Confirmed and suspected (or possible) cases	9	Argentina, Costa Rica, Belgium, Brazil, Cyprus, Denmark, Greece, South Africa, and Turkiye
Confirmed, probable, and suspected cases	7	Chile, India, Italy, Jamaica, Portugal, Spain, and UAE

1 Germany requires reporting of laboratory confirmed cases that can either (i) fulfill clinical criteria; (ii) have unfulfilled clinical criteria; or (iii) have unknown clinical picture.

2 Australia requires notification of suspected cases only to the state and territory public health units and not to the national surveillance authorities.

3 Apart from confirmed and probable cases, France requires mandatory reporting of possible (suspected) cases that are exempt from testing (criteria for exemption are—if the clinical symptoms are sufficiently suggestive, there is a context of risk of exposure to the virus, there are no signs of seriousness, and the diagnoses differentials have been ruled out).

4 Poland requires reporting of suspicious cases (after considering other causes of symptoms and considering epidemiological connections). However, when reporting to ECDC and WHO, Poland reports confirmed, suspected, and probable cases.

5 United Kingdom requires reporting of confirmed and highly probable cases (person with an orthopox virus PCR positive result in 2022 and where monkeypox remains the most likely diagnosis).

### 3.6. Case definitions for discarded cases

According to the WHO, a discarded case is a suspected or probable case for which laboratory testing of lesion fluid, skin specimens or crusts by PCR and/or sequencing is negative for Mpox (should be done on a sample other than blood specimen). Thirteen out of 32 of the investigated countries (41%) reported definitions for discarded cases or reported exclusion criteria for cases to be classified as suspected, probable, or confirmed for Mpox infection. However, of these countries only Chile and Italy had definitions that were in line with WHO recommendations (Table 5). Spain and United States remained ambiguous in terms of accepted laboratory tests (e.g., Spain does not mention sequencing for confirming cases while United States additionally accepts viral isolates from culture as described in Table 1). Brazil and Costa Rica on the other hand, mentioned only suspected cases with negative laboratory investigations as discarded case.

**Table 5:** Definition of discarded cases according to national guidelines in the investigated countries.

Country	Definition
World Health Organization (WHO)	A suspected or probable case for which laboratory testing of lesion fluid, skin specimens or crusts by PCR and/or sequencing is negative for Mpox (should be done on a sample other than blood specimen).
Austria	If the laboratory diagnostic examination of the suspected (considered same as probable) case does not provide any indication a monkeypox virus infection, the suspected case is an “excluded case” and cease any official action.
Brazil	Suspected case with negative or undetectable laboratory result for monkeypox virus (Mpox) by molecular diagnostics (real-time PCR and/or sequencing).
Chile	Suspected or probable case for which PCR and/or sequencing are negative for monkeypox.
Colombia	Probable case in which sample was taken, preserved, and processed in adequate manner for laboratory diagnosis and the result was negative.
Costa Rica	Suspected case for which laboratory tests by PCR (real-time PCR), and/or sequencing are negative in properly collected samples.
Cyprus	Upon a negative laboratory result, patients cease to be considered as outbreaks of monkeypox.
Greece	Upon a negative laboratory result, patients cease to be considered as outbreaks of monkeypox.
Italy	A suspected or probable case for which laboratory tests using PCR and/or sequencing are negative for Mpox.
Mexico	A probable case with a negative result to real-time PCR test (qPCR) or identification by sequencing that has been issued by the InDRE company.
New Zealand	A person that has been investigated and subsequently found not to meet the case definition (called as *not a case*).
Peru	A person whose cause of acute rash has been identified based on clinical diagnosis or epidemiological connection. However, to comply with the definition of probable case one must obtain and test sample for Mpox considering possible coinfection.
Spain	Suspected or probable case in which the laboratory result in samples of high quality has been negative.
United States	A case may be excluded as a suspect, probable, or confirmed case if: An alternative diagnosis can fully explain the illness OR An individual with symptoms consistent with monkeypox does not develop a rash within 5 days of illness onset OR A case where high-quality specimens do not demonstrate the presence of Orthopoxvirus or Mpox virus or antibodies to Orthopoxvirus.

## 4. Discussion

The current multi-country outbreak of the Mpox virus is a rapidly evolving situation, one that requires constant modifications and adaptations to the management and surveillance guidelines as the virus spreads. Subsequently, the WHO released interim guidelines for case recording and categorization for the member states with the aims of (i) identifying new clusters/outbreaks to provide appropriate clinical care; (ii) stopping human-to-human transmission by isolating identified cases and contact tracing; (iii) minimizing zoonotic transmission; and (iv) tailoring a coordinated global response by identifying risk groups and protecting frontline workers (13). Furthermore, the WHO has prepared a macro-enabled Microsoft Excel form that is available for member countries for data collection. The use of Go. Data platform has been recommended by the WHO for facilitation of local capture, analysis, and/or sharing of the Mpox data (13).

Although, the WHO states that the national public health authorities may adapt these recommendations and definitions based on local situation, these basic definitions for case classification were published considering the varied circumstances and capacities in all member states (13). Herein, we noticed that there are significant differences in terms of implementation and adaptation of these guidelines in the 32 investigated countries. In fact, most countries have adopted the guidelines based on the principles of ALARA (as low as reasonably achievable), something seen also during the COVID-19 pandemic (8). For example, the United States included human contact with infected animals (or products from such animals) as a criterion in the definition of a suspect case. The WHO states that this criterion is for countries with known ongoing zoonoses. However, past experience with Mpox outbreaks in the United States might have prompted such inclusion.

Case data aggregating databases such as Our World in Data (OWID; available from https://ourworldindata.org), European Centers for Disease Control and Prevention (ECDC), John Hopkins University, WHO, and others have all highlighted the discrepancies in their datasets, most of which could be traced to incomplete data collection at source and intra- and inter-country heterogeneity in guidelines, infrastructure, and indicators (16). These discrepancies are best appreciated when one investigates time series and/or comparative series. Probable causes of such ALARA adaptations stem from differences in the testing capabilities, resource scarcity, legislative delays in updating guidelines, economic capacity, insurance coverage, privacy laws etc. (8, 9). Although data modeling could be applied to overcome the ALARA heterogeneity, the very reliability of these models on previously collected data limits their accuracy and applicability in international comparisons.

Besides the ALARA adaptations, some points within the core WHO recommendations may require further consideration. For example, requiring only RT-PCR or sequencing for a confirmed case is both very limiting and not sufficiently targeted. While versatile, PCR is highly susceptible to contamination and can have varying sensitivities depending on manufacturer and end user. (17, 18). In addition, poor assay design (including primers) and sampling may result in false negative results by lowering the limit of detection (LOD) of the Mpox PCR (19). These challenges can be overcome by comparing PCR results with a previously validated and quantitated endogenous positive control. However, even this approach has its limits. Currently, the WHO guidelines require the use of a positive control that is easily detectable at low levels. But the guidelines do not ascertain what is an “easily detectable” limit (19). Furthermore, some PCR manuals describe LOD as copies/mL or copies/PCR reaction, neither of which are standardized metrics for certain clinical samples such as crusts and dry swabs (20). Although not the main goal of primary diagnostics (20), viral load quantification is still useful for future epidemiologic and standardization studies that determine LOD for designing future assays.

On the other hand, it is also plausible that Mpox PCR may produce false-positive results. There is a potential risk of cross-contamination between samples from positive and negative patients tested simultaneously in the laboratory (19). In an ideally set up PCR reaction, one would expect Cq (cycle of quantification) values to be low, an indication of high viral load in the sample. The mean Cq values for Mpox PCR tend to vary depending on sampling site (±standard deviation): 23 ± 4 Cq for skin lesions, 27 ± 7 Cq for anorectal swab specimens, and 32 ± 6 Cq for pharyngeal specimens (21). Sequencing, on the other hand, is limited by the associated downstream processing costs, technical expertise, and transportation of bulky equipment. In countries with limited experience, sequencing is hence, not the optimal method for diagnostic purposes. This may have led countries in Latin America (Argentina, Chile, Colombia, and Peru) not to include sequencing as a diagnostic criterion for confirmed cases. However, experience from the Ebola outbreak shows that relatively portable sequencing devices could be used to support epidemiological investigations in remote locations (22). We believe that the classification of cases based on laboratory findings alone may not be reasonable because of the possibility of false positives and false negatives (albeit in a small number of cases). Hence, for targeted and accurate testing and interpretation of results, it is important that clinical findings should also be included as additional criteria in confirmed case definitions.

Interestingly, parallels can be drawn between the initial approach to HIV (human immunodeficiency virus) and Mpox. Both viruses have been thought to be more transmissible among the gay, bisexual, and MSM communities. The stigma rising from the HIV epidemic is still prevalent in society, despite the implementation of countless public education programs and equal rights legislation (23, 24). In fact, a qualitative study published recently revealed that apart from fear of rejection from partners, family, and friends, intersectional stigma from healthcare providers and concerns about privacy and safety at healthcare services were equally important concerns in the MSM community (25). Since the onus for getting tested lies with the patients (based on symptom development), such associated negative perception about the neutrality of the healthcare system risks under-testing and under-reporting of the cases. Inclusion of specific criteria concerning one’s belonging to the MSM community in the probable case definition could potentially have long-term consequences. Perhaps, we think this might have led to multiple countries not adopting or dropping this criterion in their national iterations (Table 2).

In the European countries, there has been evidence of this relationship in a number of studies. The number of HIV diagnoses in the MSM community has been negatively correlated with the level of homosexual stigma (26, 27). Four of the five European countries that retained the criterion of belonging to the MSM community scored high on the Rainbow Index (RI). RI is a scoring system ranging from 0 (few rights) to 100 (well protected) that assesses legal protection, rights, access to health care, and hate speech in European countries (28). Poland was an exception, ranking in the bottom 10 of the scoring list. Contrarily, Denmark, Portugal, and Sweden were outliers, as they did not include this criterion despite their high ranking in the RI. New Zealand, on the other hand, is known worldwide for its liberal civil rights (as evidenced by its military ranking first in the LGBT Military Index). In fact, a positive correlation between the RI and the cumulative incidence of Mpox in the European countries was recently demonstrated (29).

Beyond surveillance, in clinical settings case definitions are critical for patient screening and identification that require isolation, further confirmatory testing, and contact tracing (30). For example, a turnaround time of fewer than 24 h from receipt of the specimen for PCR testing is preferred by the United Kingdom Health Security Agency (UKHSA) (31). However, experience from the US CDC shows that the median laboratory turnaround time from specimen receipt to reporting results was 30.7 h (32). Shorter turnaround times serve two purposes—first, to ensure that the patient receives appropriate care as quickly as possible, and second, to reduce the likelihood of nosocomial infection if the patient has not been properly isolated (33). Given that the family physicians/primary care providers are the first-to-detect Mpox infection in most cases (16), it is critical that doctors in such settings be properly trained and made familiar with both national and WHO case reporting guidelines.

Presently, the WHO recommends that for case identification, primary care clinics, sexual health clinics, emergency departments, dermatology clinics and other such primary care providers should employ a “simplified questionnaire and screening protocol based on the WHO case definition adapted to local epidemiology” (30), whereby adaption to the local epidemiology refers to situations when considering the differential diagnosis of infectious causes of the rash, fever, and lymphadenopathy (for classifying suspected cases). Furthermore, WHO has explicitly recommended member states test suspected cases for Mpox (using PCR/sequencing), a condition that has been adopted only by Belgium and Brazil in the national guidelines (Table 3).

The results from our comparisons also shed light on the near future challenges ahead. Selective adaptation of the guidelines—with some countries applying more stringent criteria (over-reporting) and others using more flexible criterion (under-reporting)—could lead to creation of artificial resource bottlenecks and discrepancies in the number of cases reported, thereby disproportionately demonstrating the true disease burden in the respective countries. Poor implementation of case definitions might superficially increase the number of suspected or probable cases that are reported to the healthcare system. However, upon confirmatory testing, this could lead to lesser test positivity rates and high rates of misdiagnosis, raising safety concerns (33). At the same time, this approach not only increases the demand of the confirmatory tests, but also balloon’s state expenditure for covering/subsidizing the associated costs.

According to the CDC, Mpox specimens should be handled in Biosafety Level 2 facilities. The CDC also recommends that all laboratory personnel involved in handling Mpox specimens be vaccinated against smallpox (within the past 3 years). If vaccinated personnel are not available, laboratory work may be performed in level 2 facilities, but must follow more stringent level 3 procedures (34). These requirements could also prove to tighten the testing resources available in the countries since majority of the personnel might not be vaccinated against smallpox and Level-3 procedures could take time for adoption and standardization in a Level-2 facility. This might prove to be extremely detrimental to societies applying more stringent criteria since about half of the countries have included travel to affected country as an additional epidemiological criterion in their guidelines. Though the guidelines do not mention what is meant by “affected,” it is reasonable to speculate that countries with higher reported caseloads would be considered and stigmatized. Differences in the notifiable cases (Table 4) are another example demonstrating our argument.

It is clear that the WHO guidelines need further standardization and consideration so that countries can better adapt and adjust case definitions. Indeed, publication of standardized guidelines are not enough; they merely act as a broad framework. Apart from the technical and logistical factors, attention should also be paid on access to facilities and reagents. Public opinions (religious, social, political) and education are other important factors that need to be considered. The current definitions will have implications, especially if a state considers Mpox a high-consequence infectious disease and orders a home quarantine. Unsurprisingly, analysis of the performance of COVID-19 case definitions showed that complex case definitions (multiple criteria, use of OR/AND) are doubly limited clinically. On the one hand, such definitions fail to identify those at highest risk of developing severe outcomes, while on the other hand, they fail to identify patients with common infectious symptoms such as cough and fever (33).

International coordinated collaboration and efforts are needed for sharing experiences, knowledge, and technical capabilities. This would allow for consistent reporting and surveillance recommendations, thereby harmonizing global data reporting processes and promoting a better understanding of outbreak evolution. Examples of such collaborations were seen during the COVID-19 pandemic (35). The European Observatory on Health Systems and Policies created the COVID Health System Response Monitor, a specialized tool reporting on the public health policies adopted by various countries in the WHO European region during the pandemic (36, 37). The role of non-state players and funders like Bill & Melinda Gates Foundation is equally critical for upgrading existing capabilities. While the data will be made available by the countries to the WHO and other public sources, it will also be necessary to provide incentives to legislators to facilitate the implementation of infrastructural and technological measures as well as legislation. A not-for-profit approach should be a priority, as should the provision by WHO of guarantees of fair use of the data reported. The WHO should ensure that the case of the non-authorized, third-party sharing of data is not repeated (38).

It should be noted that local adaptations of the WHO case reporting definitions are not always made on a voluntary basis, but rather out of necessity in most countries. As stated previously, WHO endorses and encourages such national iterations (13). Such an approach would certainly change the dynamics of regional and international comparisons, while ensuring effective triage of patients. It remains to be seen whether adopting separate clinical and epidemiological guidelines would facilitate and standardize the process of collecting data (33). Though similar for the majority part, United States issues two separate guidelines—clinical^1^ and epidemiological.^2^ For example, the definition for confirmed cases is similar in both guidelines except that in clinical guidelines, isolation of Mpox virus in culture from a clinical specimen is also accepted. Other noticeable difference was the definition of discarded cases which was available only in clinical guidelines but not in the epidemiological guidelines.

A similar scenario was observed for provincial definitions and case notification guidelines in neighboring Canada. British Columbia (B.C.) follows national Canadian case definitions and require notification of only confirmed and probable cases. Ontario, on the other hand, requires notification of confirmed, probable, suspected, and person under investigation. Person under investigation was defined as an individual awaiting NAAT results or an individual who does not fulfill the criteria for other case definitions.^3^

Nonetheless, our findings are constrained by certain limitations. Firstly, since the virus is currently circulating and spreading to newer countries, not all countries have released guidelines that could have helped us to get a broader picture. Secondly, the guidelines and case definitions are subject to revision as our clinical knowledge about the management of the virus evolves. As an example, in the June 2022 interim guidelines, WHO considered “hospitalized due to the illness” as a criterion for probable case, which was later dropped in the August 2022 guidelines. Yet, we noticed that Italy, Portugal, and South Africa still considered hospitalization as a valid criterion (since the national guidelines in these countries have not been updated to the August guidelines).

We assume that in the later studies, such variations would be corrected for and might not be observed. Nonetheless, we fear that these efforts would become difficult as Mpox cases decline globally. For example, the ECDC has now discontinued the publication of Mpox epidemiological reports as of 28th February 2023. Finally, we could not compare the effect of changes in case definitions with the number of reported cases. Austria and Mexico, for example, removed suspected case definitions in their national guidelines. The effects of this change are not possible to visualize since the countries notified only probable and confirmed cases. Hence, the on-ground risk–benefit analyses of these changes are hard to quantify.

## 5. Conclusion

The WHO’s guidelines for Mpox surveillance are constantly being modified and adapted to the rapidly evolving situation. In their current form, these guidelines serve more as a broad framework than a set of prescriptive rules, as evidenced by the significant variation in implementation and adaptation of these guidelines among the member states. However, these variations could be compounded when comparing provincial guidelines. Such variations arise due to the differences in testing capacity, resource constraints, legislative delays, economic resources, cultural believes, and data privacy laws. In addition, some issues within the core WHO recommendations, such as the use of RT-PCR or sequencing alone for a confirmed case, may require further consideration. False-positive and false-negative results may occur due to the limitations of PCR and sequencing. The inclusion of clinical findings as additional criteria in case definitions would allow for targeted and accurate testing and interpretation of results.

Adequate case ascertainment and reporting based on up-to-date case definitions is the cornerstone for monitoring and forecasting the global spread of the virus. The quality of data collected can be dramatically improved, fair comparisons between countries/regions can be made, and collective international public health policy can be formulated by using standardized definitions provided by the WHO. In addition, GPs should familiarize themselves with both national and WHO guidelines when reporting cases to national reporting systems.

## Data availability statement

The original contributions presented in the study are included in the article/Supplementary material, further inquiries can be directed to the corresponding author/s.

## Ethics statement

Ethical review and approval was not required for this study in accordance with local legislation and institutional requirements as all data presented in the present study has been collected from open sources and/or government resources with appropriate citations.

## Author contributions

DP and NJ conceptualized the present study and were responsible for methodology, formal analysis, and writing the original draft. DP, NJ, DK, GJ, GS, GR, MY, SV, SK, ZS, and AR were involved in data collection, validation, investigation, and revising the final draft of the paper. Visualizations were done by NJ. Project administration and supervision was done by NJ and AR. AR was responsible for resources. All authors contributed to the article and approved the submitted version.

## Conflict of interest

The authors declare that the research was conducted in the absence of any commercial or financial relationships that could be construed as a potential conflict of interest.

## Publisher’s note

All claims expressed in this article are solely those of the authors and do not necessarily represent those of their affiliated organizations, or those of the publisher, the editors and the reviewers. Any product that may be evaluated in this article, or claim that may be made by its manufacturer, is not guaranteed or endorsed by the publisher.

## Supplementary material

The Supplementary material for this article can be found online at: https://www.frontiersin.org/articles/10.3389/fpubh.2023.1178654/full#supplementary-material

Supplementary FiLe National Mpox Surveillance Guidelines.

## Footnotes

^1^https://www.cdc.gov/poxvirus/mpox/clinicians/case-definition.html

^2^https://ndc.services.cdc.gov/case-definitions/monkeypox-virus-infection/

^3^https://www.health.gov.on.ca/en/pro/programs/publichealth/oph_standards/docs/smallpox_chapter.pdf
